# The effect of moisture conditions and canal morphologies on the filling quality of iRoot SP with single-cone technique in root canals: an *ex-vivo* study

**DOI:** 10.3389/fdmed.2025.1523297

**Published:** 2025-02-20

**Authors:** Jing Yang, Xiran Xu, Jian Zhang, Kehua Que

**Affiliations:** ^1^ Department of Oral and Maxillofacial Surgery, Tianjin Medical University School and Hospital of Stomatology & Tianjin Key Laboratory of Oral Soft and Hard Tissues Restoration and Regeneration, Tianjin, Heping District, China; ^2^ Department of Oral Implantology, Tianjin Stomatological Hospital, School of Medicine, NanKai University, Tianjin, China; ^3^ Tianjin Key Laboratory of Oral and Maxillofacial Function Reconstruction, Tianjin, China; ^4^ Qingdao Stomatological Hospital Affiliated to Qingdao University, Qingdao, China; ^5^ Tianjin Medical University Institute of Stomatology, Tianjin, Heping District, China; ^6^ Department of Endodontics, Tianjin Medical University School and Hospital of Stomatology & Tianjin Key Laboratory of Oral Soft and Hard Tissues Restoration and Regeneration, Tianjin, Heping District, China

**Keywords:** iRoot SP, single-cone technique, micro-computed tomography, root canal anatomy, detine moisture

## Abstract

**Objective:**

The purpose of this study was to investigate the effects of intraradicular moisture conditions and canal morphologies on the filling quality of iRoot SP with single-cone technique in root canals.

**Materials and methods:**

Eighty-four human single-rooted premolars root canals were pair-matched with similar root canal volumes, aspect ratio (AR) curve shapes, and 3D models after micro-computed tomography (micro-CT) scanning to establish baseline consistency. Root canals were then prepared and assigned to 4 experimental groups with respect to the moisture condition tested: (1) Paper point normal drying (2) ROKEO drying (3) Paper point preliminary drying (4) Wet, followed by filling with iRoot SP single-cone technique. The effects of moisture conditions on root filling quality were studied by calculating the volume percentage of voids through micro-CT scanning and the number and score of voids under dental operating microscope (DOM) observation of the cross-sectional slices. Then classified different cross-sectional anatomical morphologies of the root canals based on the AR value and their impact on the volume percentage of voids after root canal filling were evaluated.

**Results:**

Unified the sample baseline through micro-CT pair-matching, both micro-CT and DOM provided complementary information showed that paper point normal drying and ROEKO drying displayed the lowest voids in terms of volume, number and score *(P* < 0.05) after filling with iRoot SP single-cone technique in root canals. Further analysis revealed that the voids in different segments of the root canal under four moisture conditions ranked as coronal 1/3 > middle 1/3 > apical 1/3 (*P* < 0.05). In addition, the cross-sectional AR value of the root canal was positively correlated with the volume of voids within each moisture condition(*P* < 0.05).

**Conclusion:**

The intraradicular moisture conditions and cross-sectional anatomical morphology had significant effect on the filling quality of iRoot SP with single-cone technique.

## Introduction

Root canal treatment (RCT) is primarily performed to seal the root canal system. In preventing tissue fluid, bacteria, and/or their products from penetrating the root canal and causing re-infection after root canal preparation (1), various root canal filling materials, techniques, and sealers have been developed. At present, different types of root canal sealers are applied in clinical practice. In particular, bioceramic sealers are becoming increasingly popular because of their excellent biocompatibility and biological activity, non-toxic side effects, and promotion of repair performance. EndoSequence BC (Brasseler, Savannah, USA), also known as iRoot SP (Innovative Bioceramix, Vancouver, Canada), is an insoluble, hydrophilic, ready-to-use calcium silicate-based root canal sealer composed of zirconium oxide, calcium silicates, calcium phosphate, calcium hydroxide, filler, and thickening agents (2). This sealer is usually stored in an airtight syringe, and it can be directly applied into the root canal space (3, 4). With the widespread development and application of iRoot SP, the single-cone filling technique is also becoming widely used, which is simple, easy to learn and perform, and less time consuming, and it can improve adaptation to the dentine wall (5–8). The filling quality of iRoot SP combined with the single-cone technique has gradually attracted the attention of researchers and clinical doctors.

Different moisture conditions in the root canal have a remarkable impact on the sealing properties and adhesion of root sealers (9). Normally, the dentine tubules are saturated with water, unless the teeth are thoroughly dried (10). The setting reaction of hydrophilic iRoot SP is initiated by the presence of water in the dentine tubules, and it produces hydroxyapatite. This procedure enhanced the penetration of iRoot SP into the dentine tubules, facilitating the formation of micromechanical locks and a chemical bond between the sealer and the dentine wall (11–13). However, it still falls short in effectively displacing water in wet root canals, which can result in bond disruption and an increase in voids (9, 13). If the voids are adjacent to the inner surfaces of the root canal, dead spaces will form and the success of the treatment will be reduced due to possible microleakage and infection (14). According to the manufacturer's recommendation, keeping the root canal wall moist is beneficial for the sealing performance of iRoot SP, but the control of ideal moisture conditions and clinical steps have not been standardized (9, 11, 13, 15). Once the dentists cannot measure the moisture or the dryness inside the root canals during endodontic treatment, the canal drying instructions should be specific and direct to be systematically reproduced in clinical situations (16).

Anatomical matching of root canal morphology should be regarded as the first underlying experimental step of any comparative *in vitro* study in endodontics (17). Root canals have an irregular anatomical morphology, including oval, long-oval, and flattened cross-section, which may affect thorough debridement and then cause voids after filling (18), as well as the comparison results among the experimental groups, especially in small sample sizes. Recent studies began to use a novel methodology for matching baseline based on anatomical parameters through micro-computed tomography (micro-CT) scanning (3, 17). It is a nondestructive and visual method to calculate the volume of filling material without damaging the specimen and provide a 3D assessment of the filling quality, which makes it possible to identify areas of failure and voids.

Therefore, considering the search for a clinical method to control water content, reduce the porosity, and improve the quality after root filling, this study aimed to investigate the effect of moisture conditions on the voids of iRoot SP combined with the single-cone technique in root canals with various anatomical morphologies. The calculation was assessed by using micro-CT analysis of scanning image data and dental operating microscope (DOM) observation of cross-sectional slices to verify the validity of the methods. In addition, we evaluated the effect of the different cross-sectional anatomical morphologies of root canals on the volume percentage of voids after root canal obturation.

## Material and methods

**Figure 1:**
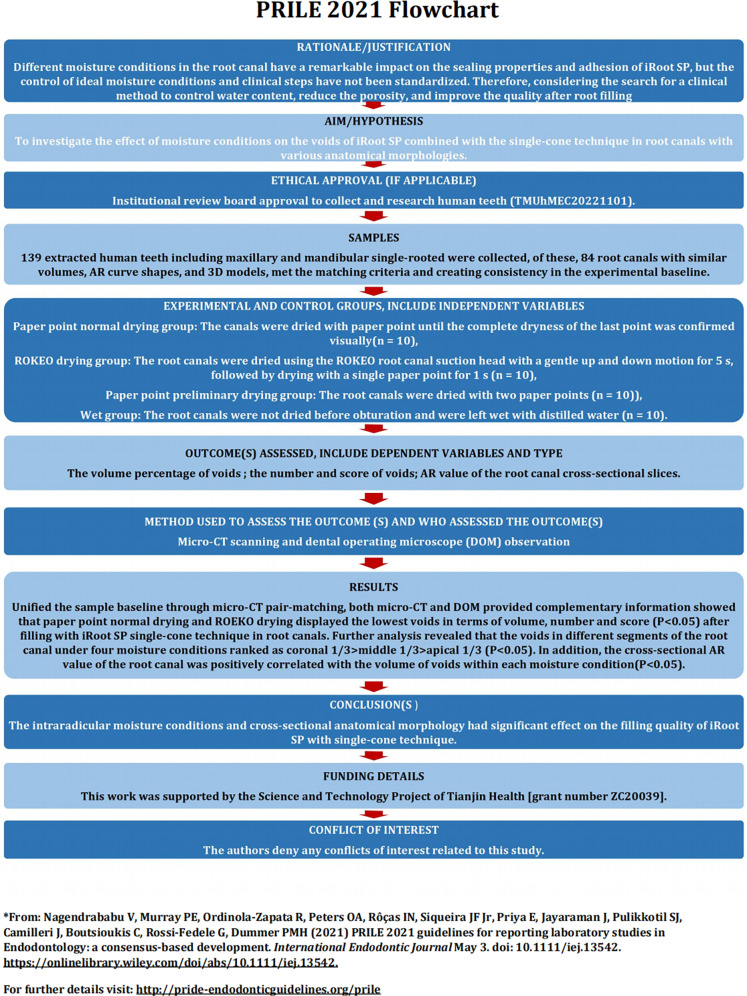
Flowchart according to the preferred reporting items for laboratory studies in endodontology (PRILE) 2021 guidelines.

The manuscript of this laboratory study has been written according to Preferred Reporting Items for Laboratory studies in Endodontology (PRILE) 2021 guidelines (19). The PRILE 2021 flowchart is presented in Figure 1.

### Sample size estimation

The number of teeth required to verify significant differences between the groups was estimated on the basis of the study of NAGAS E (9) in which an effect size of 0.91 was input together with an alpha-type error of 0.05 and power beta of 0.8 into an independent t-test family procedure (Power and Precision software, Biostat, Englewood, NJ). The output indicated a minimum of five teeth per group, which can obtain the difference in the impact of different root canal moisture conditions on root filling quality. Therefore, a total of 21 teeth per group was selected for the present study.

### Specimen selection and grouping

This study was approved by the ethics committee of the Stomatological Hospital of Tianjin Medical University, Tianjin, China (TMUhMEC20221101). A total of 139 extracted human teeth including maxillary and mandibular single-ro oted premolars with intact pulp chamber bottom and fully formed apices, extracted for periodontal or orthodontic reasons, were collected from the Maxillofacial Surgery Clinic of the Stomatological Hospital of Tianjin Medical University, Tianjin, China. The periodontal membrane and dental calculus were removed, and the teeth were stored in 0.5% thymol solution in a 4°C refrigerator before use. The criteria for teeth selection were as follows: (1) Teeth with no evident cracks on the root surface or incomplete root formation were selected after observation under DOM. (2) Single-rooted premolars were selected without root canal treatment, calcification, or root resorption.

Initially, 93 specimens were selected for the study. Then, scans were performed (SkyScan 1276; Bruker MicroCT, Belgium; 70 KV, 114 mA, pixel size of 17 *μ*m, 180° rotation with a rotation step of 0.5°, 0.5 mm-thick aluminum filter, exposure time 686 ms). Air calibration was performed before each scan to minimize ring artifacts. After scanning, NReconv. 1.7.3.1 (Bruker micro CT) was used for three-dimensional (3D) reconstruction, with 40% beam hardening correction and 10% ring artifact correction. After excluding teeth with complex anatomical structures such as multiple root canals and accessory canals, we used CTAn v.1.18.1 (Bruker micro CT) to determine the grayscale range for each sample on the grayscale histogram. To ensure segmentation accuracy, we compared the original scanned image and the binary image by limiting contrast (20). Furthermore, the volume (mm^3^) of the root canal was calculated.

**Figure 2:**
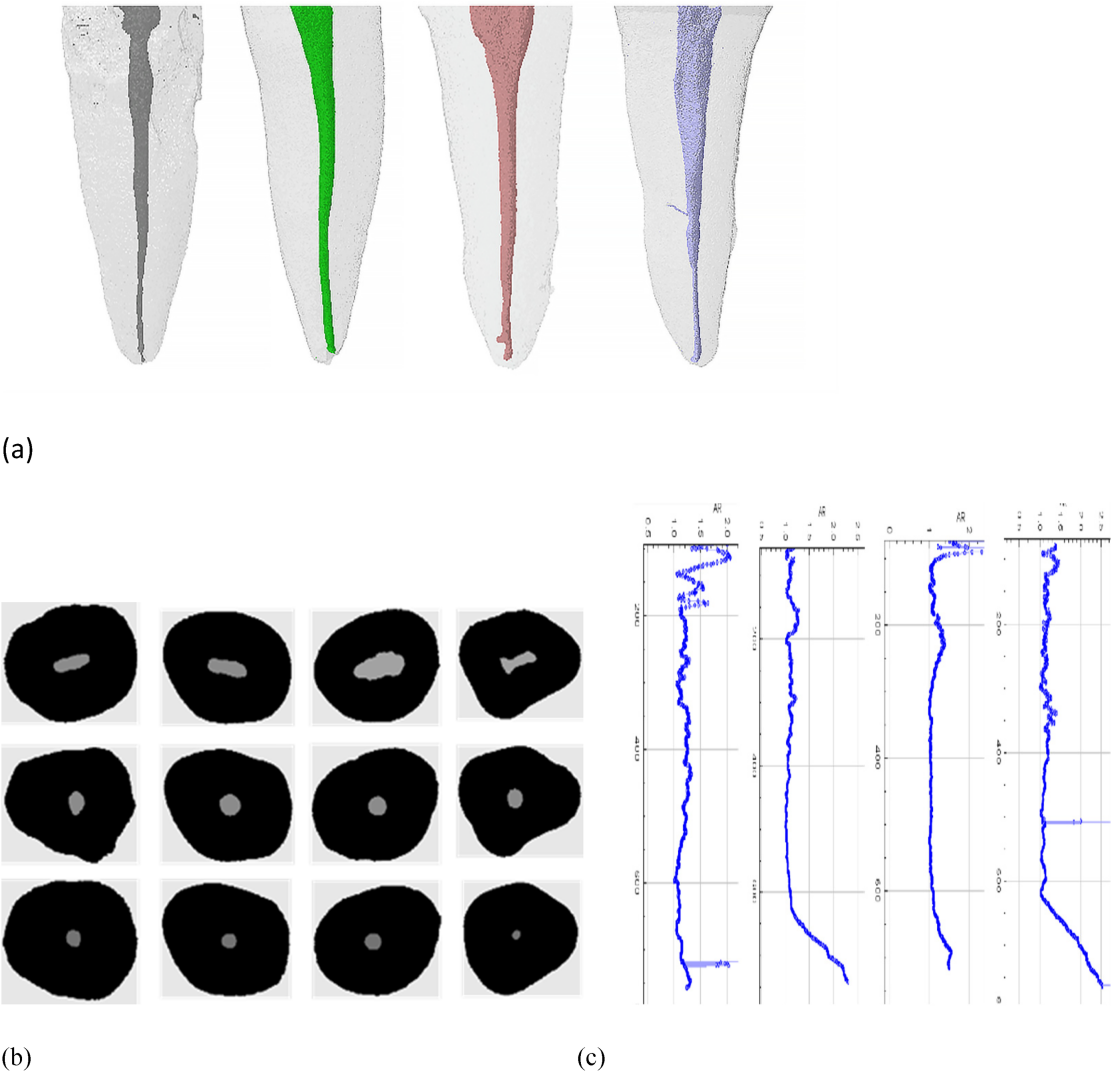
Representative sample matching process: **(a)** 3D model of similar tooth roots and root canals, **(b)** cross-sectional images of similar root canals after Mirco CT scan reconstruction, **(c)** AR value curve of similar root canals.

The aspect ratio (AR) of each cross-section of the root canal was calculated using ImageJ (Fiji v.1.51n; Fiji), and the resulting data were plotted into a curve. The AR is defined as the ratio of the major to the minor diameter. Then, Ctvol 2.3.2.0 (Bruker microCT) was used to create a 3D model of the root canal. Figure 2 shows the matching process of the sample: identifying root canals with similar root canal volumes, AR curve shapes, and 3D models, creating consistency in the experimental baseline. After applying these strict inclusion criteria, a total of 84 samples (*n* = 84) met the matching criteria, and they were divided into four experimental groups (*n* = 21).

### Root canal preparation

A high-speed diamond bur was used to remove a portion of the crown from the coronal plane, with a uniform sample length of 16 mm. Subsequently, a size #10 K-File was inserted into the root canal until the tip was just visible beyond the apex under DOM. The working length was determined by subtracting 1 mm from this length. Then, the root canals were enlarged using the ProTaper Universal (PTU, Dentsply Sirona, Switzerland) nickel titanium system until the file F3 (30# 09) reached the working length. Between each file size, the root canals were irrigated with 5 ml 3% sodium hypochlorite (NaOCl) using a 31 # lateral opening needle. After preparation, the root canals were irrigated with 17% EDTA for 1 min, followed by rinsing with 10 ml of distilled water to remove all chemicals (13).

The specimens with consistent anatomical morphological baseline were randomly assigned to the following four experimental groups to evaluate the impact of different intracanal moisture conditions on root filling quality before obturation:

(1) Paper point normal drying group: The canals were dried with paper point until the complete dryness of the last point was confirmed visually.
(2) ROKEO drying group: The root canals were dried using the ROKEO root canal suction head with a gentle up and down motion for 5 s, followed by drying with a single paper point for 1 s.
(3) Paper point preliminary drying group: The root canals were dried with two paper points.
(4) Wet group: The root canals were not dried before obturation and were left wet with distilled water. We used 3002# paper points (Absorbent Paper Points, GAPA,China)for drying.

### Root canal filling

The root canals were filled with PTU F3 gutta-percha point (Dentsply, Switzerland) and iRoot SP using the single-cone technique. According to the manufacturer's recommendation, the canals were first fully injected with iRoot SP via a syringe tip. Then, the tip was slowly pulled from the canal's engagement point towards the orifice.

Subsequently, a PTU F3 gutta-percha point modified to the working length with sealer, was slowly inserted into the canals. A heat plugger (B&L, USA) was used to cut excess filling material at the orifice level, and the cone was vertically condensed with a cold plugger (Coltène, Switzerland). The coronal seal was temporarily sealed with Cerviton (GC, Japan). Thereafter, the specimens were stored at 37°C and 100% humidity for 1 week to ensure a complete set of the materials.

### Micro-CT analysis

Before and after root filling, scans with air calibration were performed (SkyScan 1276; Bruker MicroCT, Belgium; 70 KV, 114 mA, pixel size of 15 *μ*m, 360° rotation with a rotation step of 0.5° and average frame of 2, 0.5 mm-thick aluminum filter, exposure time 500 ms). Then, the images obtained were reconstructed and quantified using the above mentioned methods. The root canals were divided into three segments at a distance of 4, 8, and 12 mm from the apical part. The volume percentage of voids in each third was measured and calculated. The volume percentage of voids (V%) is calculated as follows:

Void volume percentage (V%) = [1—(filling material volume/root canal volume)] × 100%

**Figure 3:**
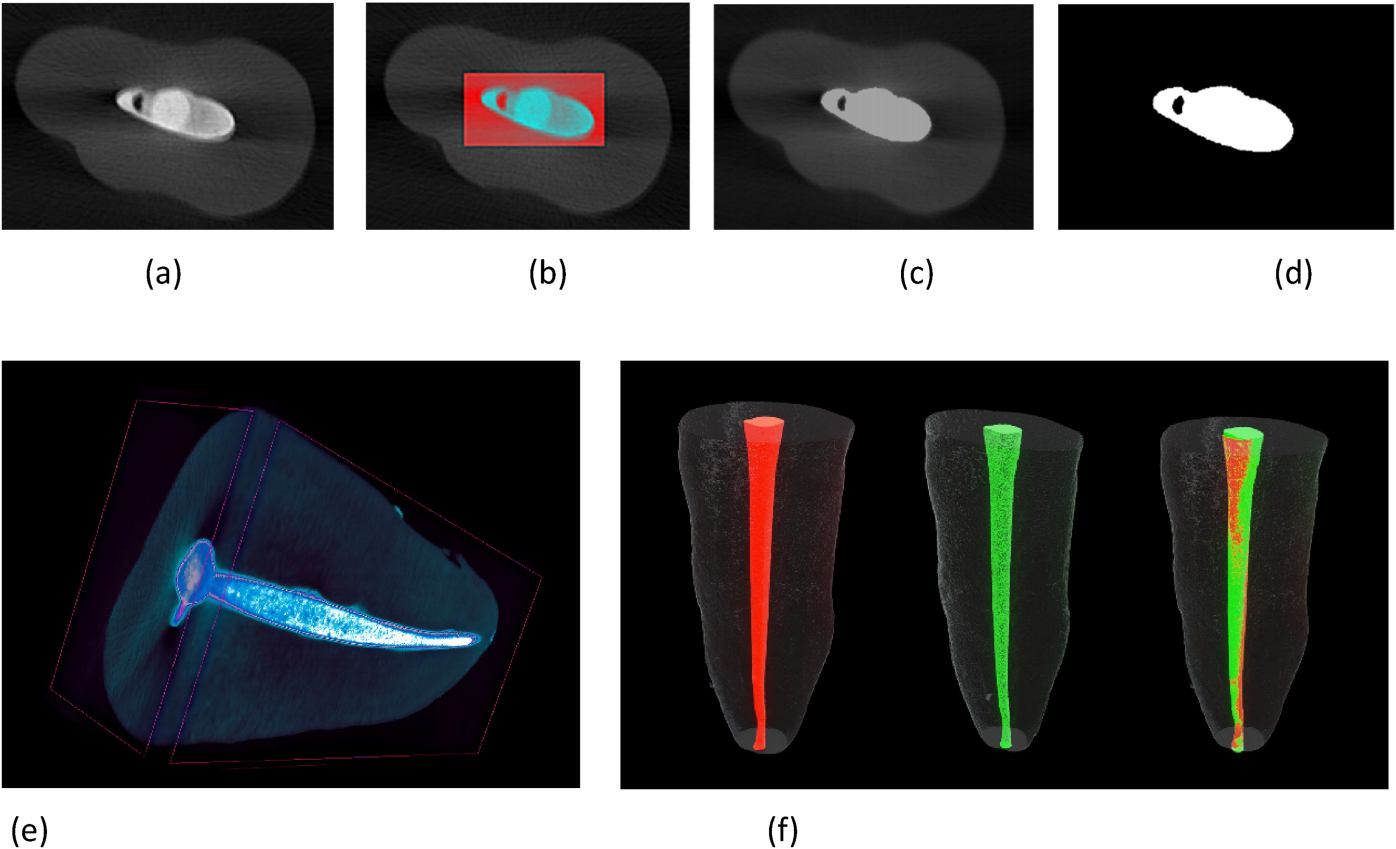
mirco-CT scan reconstruction images show voids **(a)** cross-sectional images, **(b)** selection of region of interest, **(c)** automatic segmentation, **(d)** binarization removal of dentine, **(e)** reconstruction of 3D model after root filling, **(f)** registration of 3D model before and after root filling: red, after root canal preparation; green, after root filling.

3D slicer 5.0.3 software was used to match the 3D models before and after filling with each other along the long axis of the root canal and to evaluate the quality of RCT. By quantifying the difference, the RCT material and voids were visualized. In this study, areas without filling material within the root canal space post-filling procedures were defined as voids (21). Notably, this study examined only the volume of the prepared root canal, filling materials, sealers, and voids, excluding the accessory root canal or any lateral root canal and isthmus Figure 3.

**Figure 4:**
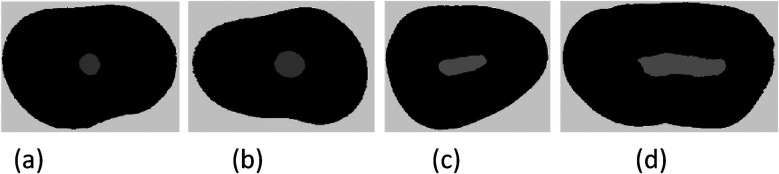
Schematic diagram of cross-sectional anatomical morphology of root canal **(a)** circular root canal AR = 1, **(b)** oval root canal l AR < 2, **(c)** long oval root canal 2 ≤ AR < 4, **(d)** flattened root canal AR ≥ 4.

Moreover, a study conducted by Jou et al. (22) has defined the anatomical morphology of the root canal cross-section based on the AR value (Table 1). The coronal, middle, and apical one-third of the root canal were defined as circular, oval, long oval, and flattened root canals, respectively (Figure 4), and then the impact of cross-sectional anatomy on the filling quality of the root canal was evaluated by determining the percentage of void volume in each segment of the root canal.

**Table 1:** Classification and definition of root canal anatomical morphology (AR value).

Classification	Definition
Circular root canal	AR = 1
Oval root canal	AR < 2
Long Oval root canal	2 ≤ AR < 4
Flattened (flat, ribbon)	AR ≥ 4
Irregular	cannot be defined by 1–4

### DOM observation

**Figure 5:**
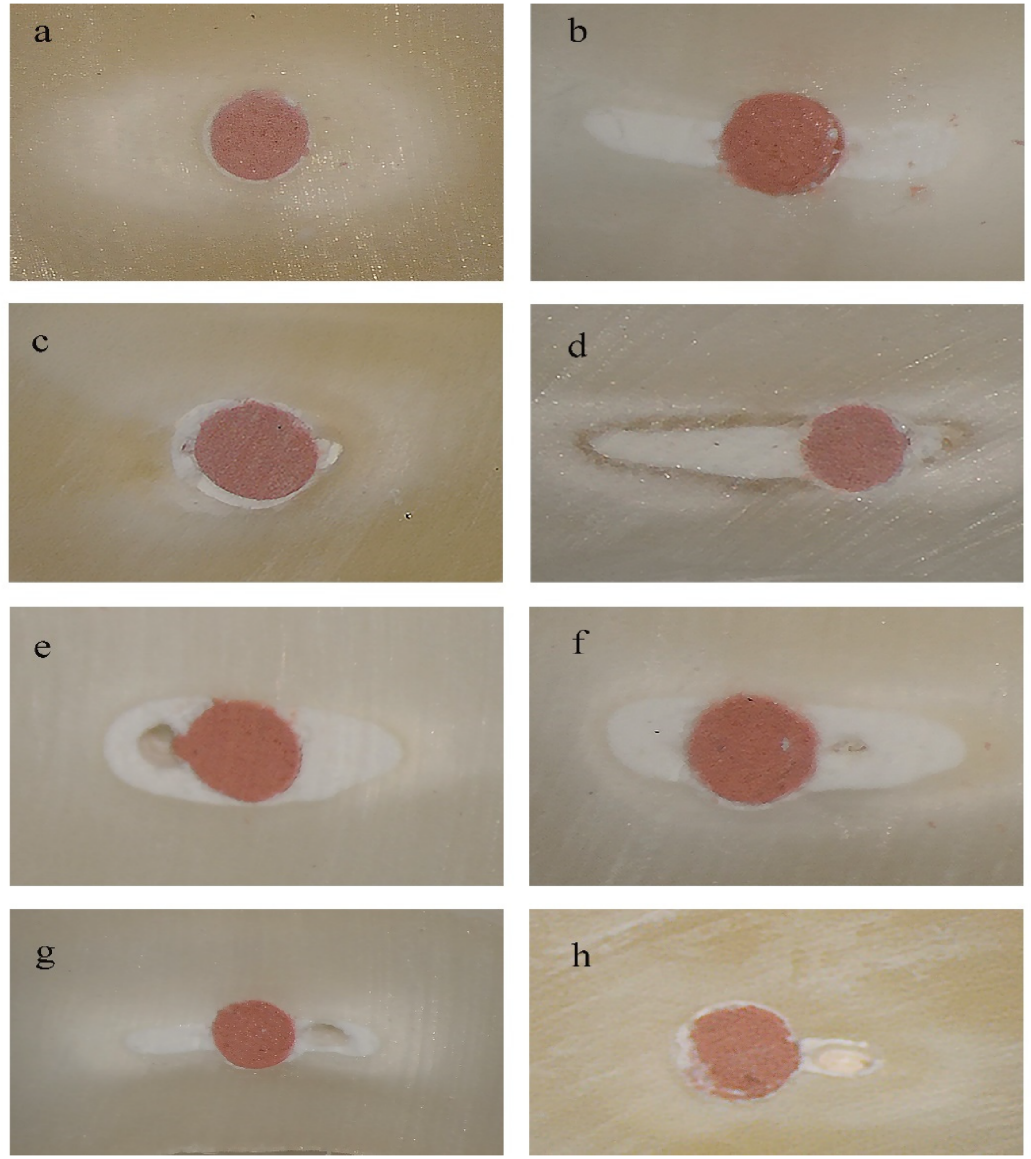
Representative images observed under DOM (original magnification 16×): **(a,b)** score 1 from paper point normal drying group, **(c,d)** score 2 from ROEKO drying group, **(e,f)** score 3 from paper point preliminary drying group, **(g,h)** score 4 from wet group.

After micro-CT analysis, a diamond-coated saw was used to obtain every two slices with a thickness of 1.5 ± 0.1 mm section from the coronal, middle, or apical part, perpendicular to the longitudinal axis of the root. The pictures of each cross-sectional slice were taken using DOM at 16× magnification (Figure 5). The number of voids was counted and scored using the scoring system described in Table 2 (23). The scoring system was evaluated by two independent observers who were unaware of the moisture conditions of each specimen slice. For each section, measurements were repeated two times, and the mean was calculated.

**Table 2:** Scores used for evaluating the filling quality by void detection.

Score	Definition
1	Well-condensed filling that showed only a few minor voids (less than 0.1 mm in diameter).
2	An imperfectly condensed filling that showed some minor voids (more than 3 defects) or medium-sized voids (0.1 mm to 0.2 mm in diameter).
3	Inadequately condensed filling that showed many minor voids (more than 5 defects) or large voids (more than 0.2 mm in diameter).
4	Poorly condensed filling that showed many minor voids (more than 7 defects) or empty space connecting separate canal walls.

### Statistical analysis

The experimental data was statistically analyzed using SPSS 26.0 (SPSS Inc, Chicago, IL). The normality of variable distribution was evaluated through the Shapiro–Wilk test. After filling, the percentage of void volume and the AR value of the root canal cross-section did not meet the normal distribution. Therefore, the Kruskal–Wallis test was used, and pairwise comparison was conducted. The number and score of voids in each group of sample slices followed a normal distribution. One-way ANOVA was used to compare the mean and standard deviation between the groups, and the Turkey multiple comparison test was used to further analyze the differences. The statistical significance level was set at *P* < 0.05.

## Results

### Micro- CT analysis

**Figure 6:**
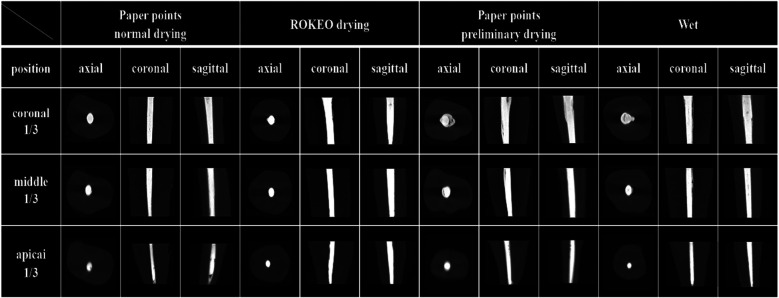
Representative Micro-CT images of premolar root canals in axial, coronal, and sagittal positions after iRoot SP single-cone filling.

Overall, non-homogenously distributed voids were found in each segment of the root canal after root filling under four moisture conditions (Figure 6).

A summary of the mean percentage ± standard deviation of the volume percentage of voids in each group after RCT is presented in Table 3. The results of the Kruskal–Wallis Test indicated that the paper point normal drying group and ROEKO drying group had significantly lower volume percentage of voids compared with the wet group (*P* < 0.05), whereas no significant differences were observed among the other groups (*P* > 0.05).

**Table 3:** The volume percentage of voids after root filling under 4 moisture conditions.

				Kruskall-Wallis Test	
Segment	Group	*n*	Mean ± SD	Pairwise comparison	*P*
Coronal	Paper points normal drying	21	8.53 ± 3.02	1–2	0.222
	ROKEO drying	21	9.55 ± 3.01	1–3	0.141
	Paper points preliminary drying	21	10.05 ± 3.45	1–4	0.002*
	Wet	21	11.72 ± 3.47	2–3	0.803
				2–4	0.067
				3–4	0.114
Middle	Paper points normal drying	21	5.34 ± 3.10	1–2	0.552
	ROKEO drying	21	4.81 ± 2.83	1–3	0.494
	Paper points preliminary drying	21	6.55 ± 4.35	1–4	0.008*
	Wet	21	7.94 ± 3.12	2–3	0.201
				2–4	0.001*
				3–4	0.048*
Apical	Paper points normal drying	21	4.33 ± 2.43	1–2	0.586
	ROKEO drying	21	4.11 ± 3.07	1–3	0.651
	Paper points preliminary drying	21	5.42 ± 4.38	1–4	0.003*
	Wet	21	7.58 ± 3.05	2–3	0.319
				2–4	0.000*
				3–4	0.011*
Overall	Paper points normal drying	21	7.30 ± 2.76	1–2	0.820
	ROKEO drying	21	7.37 ± 2.77	1–3	0.226
	Paper points preliminary drying	21	8.57 ± 3.37	1–4	0.002*
	Wet	21	10.05 ± 3.08	2–3	0.325
				2–4	0.005*
				3–4	0.054

Pairwise comparison: 1- Paper points normal drying, 2- ROKEO drying, 3-Paper points preliminary drying, 4-Wet.

* Indicates *P* < 0.05.

Further analysis revealed that the volume percentage of voids in different segments of the root canal under four moisture conditions ranked as coronal one-third > middle one-third > apical one-third (*P* < 0.05). Moreover, in coronal one-third, the paper point normal drying group displayed a significantly lower volume percentage of voids than the wet group (*P* < 0.05). In the middle and apical one-third, the wet group showed the highest volume percentage of voids (*P* < 0.05). Nevertheless, no significant differences were observed among the other groups (*P* > 0.05).

**Figure 7:**
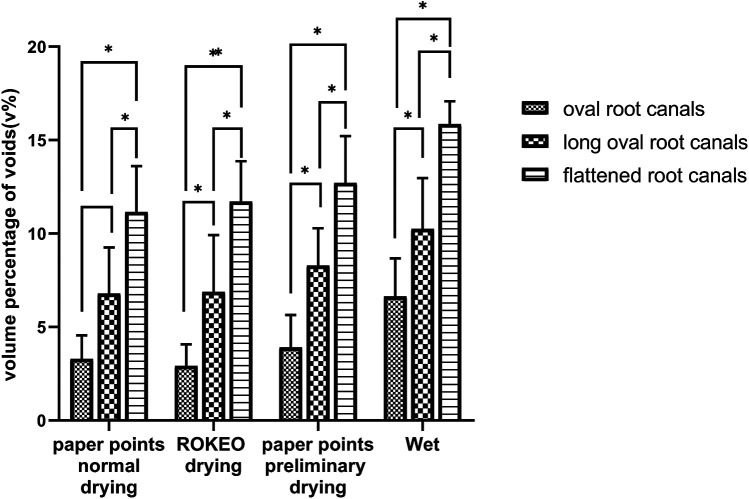
The effect of cross-sectional anatomical morphology of root canals on the volume percentage of voids after root filling. *Indicates *P* < 0.05.

Figure 7 shows that the volume percentage of voids after RCT was significantly impacted by the cross-section AR value of the root canal (*P* < 0.05). When comparing the volume percentage of voids within each moisture condition, oval (AR < 2) root canals had the lowest percentage of voids compared with long oval (2 ≤ AR < 4) root canals and flattened (AR ≥ 4) root canals (*P* < 0.05).

### DOM observation

Table 4 shows significant differences with regard to the average number of void and void scores across various groups (*P* < 0.05). The paper points normal drying and ROEKO drying groups had fewer voids and void scores than the other groups (*P* < 0.05).

**Table 4:** The number of voids and void score results of sample slices in each group (Mean ± SD).

Group	Coronal	Middle	Apical	Overall
Number of Voids				
Paper points normal drying	3.79 ± 1.63^b^	2.43 ± 1.60^c^	0.64 ± 0.63^c^	2.29 ± 1.86^c^
ROKEO drying	4.21 ± 1.58^b^	2.43 ± 1.34^c^	1.21 ± 0.89^bc^	2.62 ± 1.78^bc^
Paper points preliminary drying	4.86 ± 1.99^ab^	3.57 ± 1.16^ab^	2.43 ± 1.22^ab^	3.62 ± 1.78^ab^
Wet	6.14 ± 2.58^a^	3.71 ± 1.64^a^	3.29 ± 2.67^a^	4.38 ± 2.60^a^
Score of Void				
Paper points normal drying	2.29 ± 0.61^B^	1.50 ± 0.52^C^	1.14 ± 0.63^B^	1.64 ± 0.69^B^
ROKEO drying	2.29 ± 0.91^B^	1.86 ± 0.66^BC^	1.36 ± 0.50^B^	1.83 ± 0.79^B^
Paper points preliminary drying	2.93 ± 0.92^AB^	2.43 ± 0.85^AB^	2.21 ± 1.05^A^	2.52 ± 0.97^A^
Wet	3.57 ± 0.51^A^	2.79 ± 0.80^A^	2.43 ± 0.94^A^	2.93 ± 0.89^A^

Note: Different lowercase letters indicate statistical differences in the number of voids between groups (*P* < 0.05), while different uppercase letters indicate statistical differences in void scores between groups (*P* < 0.05).

Furthermore, when comparing different segments of the root canal under four moisture conditions, the results from high to low ranked as coronal one-third > middle one-third > apical one-third (*P* < 0.05). Meanwhile, a lower average number of void and void scores in each segment of the root canal were observed in the paper point normal drying group and ROEKO drying group than the wet group (*P* < 0.05). In addition, the paper point normal drying group and ROEKO drying group were significantly lower than the paper point preliminary drying group in the middle and apical one-third (*P* < 0.05). No statistically significant differences were found among the other groups (*P* > 0.05).

## Discussion

In this study, micro-CT and DOM were used to evaluate the impact of four moisture conditions on the filling quality of RCT combined with the single-cone technique of iRoot SP. Considering minimizing the impact of anatomical morphological changes and providing a consistent baseline for root canal laboratory studies, pair-matched experimental groups based on similar volume, AR graphical curves, and 3D models of the root canals were performed in this study (17). Based on four common clinical root canal moisture conditions, ROKEO drying was selected as the representative negative pressure suction product because of its simplicity, convenience, and widespread usage. In addtion, the specialized negative pressure devices are not commonly used in China and many other countries as their complexity and higher cost; the other three conditions were closely related to clinical practice by simulating the simple drying methods commonly used. Furthermore, to evaluate the differences in root canal moisture condition and anatomical morphology, all samples were filled using iRoot SP combined with the single-cone technique to standardize obturating processes and reduce variable errors (13).

Voids and gaps are unfilled root canal spaces which may harbor viable bacteria and serve as a pathway for microleakage (21). In contrast to previous research (24, 25), our study also investigated the impact of the anatomical morphology of root canals on the filling quality of iRoot SP, employing micro-CT technology for analysis. As the root canal structure is intricate and changes at various levels throughout the whole root canal (18, 25, 26), the cross-sectional AR value of the root canal was positively correlated with the volume of voids. Moreover, the voids gradually increased from the apical part towards the coronal third possibly because the root canal tends to be more regular at the apical region (27, 28). Therefore, the more irregular of the root canal anatomical morphology, the more relative is the space proportion of the sealer, and the easier the voids appeared in the single-cone technique. Although bioceramic sealers such as iRoot SP are hydrophilic and can achieve relatively higher strength, achieving good sealing in the apical third of significantly irregular root cannal, could still be difficult. Therefore, the combination of utilizing accessory points or warm vertical compaction could be the best approach for achieving optimal root canal filling to effectively fill long-oval or flattened root canals in the coronal and middle third using the iRoot SP single-cone technique. This study does have certain limitations. The complexity and variability of root canal morphology make it challenging to achieve complete balance and consistency, which can hinder the implementation of standardized treatment protocols in clinical practice. Furthermore, as clinical cases involve inherent variability, root canal morphology cannot be fully standardized. Consequently, long-term follow-up is essential for assessing single-point filling using biomaterials.

The present research on the impact of moisture conditions on filling quality focused on dye-leakage study and push-out test, but inner air in root canals could hinder dye penetration (29, 30). In this study, notably, the two analysis methods showed different results when comparing voids between the paper point preliminary drying and other groups. Based on the hypothesis of Jung and Yeon et al., such a difference could be due to the fact that radiopaque sealers (Cone-beam Artifacts) affected the detection of voids within the root filling via micro-CT (23, 31). Meanwhile, material stripping during sectioning by DOM observation was a destructive method for samples, which could lead to an increase in porosity (23, 32). Therefore, micro-CT and DOM could be used to provide complementary information in analyzing the quality of root canal obturation.

The current research methods for controlling water content in the root canal vary with one another, and such methods could result in different dentine moisture levels (33). In this study, apart from using paper point normal drying, ROKEO drying was used, which works similarly to the Luer vacuum adapter by utilizing negative pressure to dry the root canal. Our findings indicated that the abovementioned methods achieved better results with regard to the filling quality of RCT, which were consistent with the findings reported by Zmener and Nagas (9, 13). These two drying methods can remove significant water while still keeping moisture in the dentine wall, which can enhance the setting reaction of hydrophilic iRoot SP and reduce voids after oburation (13). Conversely, wet conditions resulted in the lowest filling quality, which was consistent with previous studies (9, 11, 13, 34). This result may be due to the fact that hydrophilic iRoot SP cannot completely displace water during the polymerization, and residual water in the root canal might result in bond disruption and void formation (13). Given the lack of patience or recognition of the impact of moisture on root canal filling by dentists, preliminary drying may occur frequently in clinical practice, and drying may be stopped without confirming the absence of significant moisture in the root canal. Despite variations in experimental methodology, specimen selection, pair-matching standards, and materials between our study and previous research, the outcome showed similarities, confirming that maintaining the dentine wall moist instead of excessive drying or over-wetting in clinical procedures can improve the filling quality of RCT.

Because of the complexity of root canal anatomy, It is difficult to achieve sealing of the root canal system. Studies have shown that the expansion of gutta-percha may improve the filling quality (35), moisture's presence may also result in expansion and decrease leakage caused by sealer dissolution (36). At present, there are few studies on the expansion rate of root canal sealer, the relationship between sealer and dentin interface is mainly reflected by the bond strength test (9, 13). In this study, the volume of voids was identified mainly by micro-CT scanning, the expansion of the sealer after absrobing water has a certain impact on the results of this study, but it is relatively small, we will further consider this issue in future experiments.

No filling technique has been shown to achieve completely void-free root canal fillings (21). Research indicates that in the coronal third of the canal, continuous wave of condensation technique demonstrates a significantly lower percentage of voids compared to single-cone technique (7, 37). Furthermore, some studies suggest that single-cone technique provides better filling quality in narrow round root canals (38). And in oval root canals, several researchers argue that warm vertical compaction technique yields a lower percentage of voids than both cold lateral compaction technique and single-cone technique (21, 39). Other studies report there were no significant differences in the impact of various root canal filling techniques on void after filling (40). More studies are required to confirm the relevance of the present results.

## Conclusions

Achieving a complete absence of voids after root filling remains a challenge, regardless of the drying method used. Since the degree of dryness cannot be quantitatively assessed, this study indicated that normal drying with paper points and ROKEO drying were associated with lower percentages of void volume, fewer voids, and lower void scores. When visually verifying the dryness of the paper points, it is advantageous to minimize voids following the root filling process. However, significantly irregular root canals, especially when using the iRoot SP single-cone technique, posed challenges for achieving tight root canal fillings. To achieve optimal results, a combination of utilizing accessory points or employing warm vertical compaction may be the most effective approach.

## Data Availability

The original contributions presented in the study are included in the article/Supplementary Material, further inquiries can be directed to the corresponding authors.
